# Epidural Hematoma Following Computed Tomography (CT) Myelography With Continued Antithrombotic Therapy: Two Cases Demonstrating Full Neurological Recovery

**DOI:** 10.7759/cureus.84463

**Published:** 2025-05-20

**Authors:** Koji Suto, Atsuyuki Kawabata, Tatsuhiro Kaku, Kazuo Kusano, Kazuyuki Otani, Shigeo Shindo, Toshitaka Yoshii

**Affiliations:** 1 Department of Orthopedics, Kudanzaka Hospital, Tokyo, JPN; 2 Department of Orthopedics, Tokyo Medical and Dental University, Tokyo, JPN

**Keywords:** complications of anticoagulation, ct myelography, lumbar, spinal epidural hematoma (seh), spine

## Abstract

Epidural hematoma after computed tomography (CT) myelography is a rare but potentially devastating complication. We report two elderly patients who developed epidural hematomas shortly after undergoing CT myelography, despite continuation of antiplatelet or anticoagulant therapy. In both cases, emergent decompression surgery was performed within six hours of symptom onset, resulting in complete neurological recovery. These cases underscore the importance of prompt recognition and intervention and suggest that continuation of anticoagulation may be feasible under close monitoring in select patients.

## Introduction

Spinal epidural hematoma (SEH) is a rare but potentially devastating complication that can lead to acute neurological deterioration. While SEH is more commonly associated with trauma, spinal procedures, or anticoagulant therapy, its occurrence following diagnostic myelography is extremely uncommon and scarcely reported in the literature. The precise incidence of SEH after myelography remains unknown, but several risk factors, such as advanced age, coagulopathy, and the use of antithrombotic agents, have been implicated in similar iatrogenic spinal hematomas [1,2]. Although myelography is generally considered a safe diagnostic tool, it is not without risks. Reported complications include postlumbar puncture headache, infection, and, in rare cases, epidural hematoma or seizures [3]. Current guidelines recommend temporary discontinuation of anticoagulants and antiplatelet drugs before invasive spinal procedures to reduce the risk of hemorrhagic complications [4]. However, in clinical practice, these protocols are not always followed, particularly when myelography is deemed low risk. In this report, we present two cases of SEH that developed shortly after myelography was performed without interruption of antithrombotic therapy. These cases highlight the importance of periprocedural vigilance to enable early recognition and management, which may help prevent irreversible neurological deterioration.

## Case presentation

Case 1

A 79-year-old woman presented with persistent severe back pain and gait disturbance two months after being treated conservatively with a brace for an L2 compression fracture sustained in a fall. Initially diagnosed at a local hospital, she visited our facility due to progressive kyphotic alignment and worsening symptoms. Upon initial evaluation, physical examination and blood tests were performed. Her medical history included cerebral infarction (for which she was on aspirin), lung cancer, appendicitis, hypertension, and hypothyroidism. Laboratory results showed a platelet count of 164,000/μL, prothrombin time international normalized ratio (PT-INR) of 0.99, and activated partial thromboplastin time (APTT) of 29.1 seconds.

Neurological examination revealed mild motor weakness: Medical Research Council (MRC) grade 4/4 in the flexor hallucis longus (FHL) and 4/5 in the extensor hallucis longus (EHL). Sensory function was intact, with no bladder or bowel dysfunction and normal deep tendon reflexes. Radiography revealed delayed union at the T8 vertebra, and magnetic resonance imaging (MRI) demonstrated mild cord compression at the same level. The patient was admitted for further evaluation, and myelography was performed using a 20-G needle. The patient was on low-dose aspirin due to a history of myocardial infarction and coronary stenting. The prescribing physician deemed discontinuation to carry a significant thromboembolic risk, and thus, aspirin was continued with careful monitoring during the procedure.

**Figure 1:**
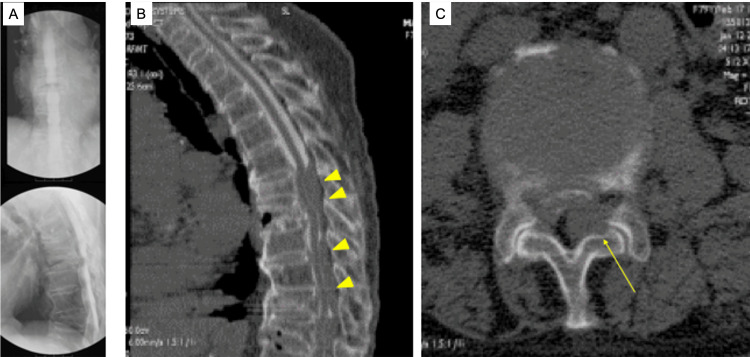
Radiographic findings in Case 1 after myelography. (A) Lateral X-ray during myelography showing dorsal pooling of contrast at the thoracic level. (B) Sagittal CT myelography demonstrating a dorsal contrast defect extending from T7 to T12 (yellow arrowhead). (C) Axial CT myelography image showing dorsal compression of the thecal sac by an epidural mass (yellow arrow) CT: computed tomography

Approximately two hours after the procedure, she developed acute lumbar pain followed by progressive paraplegia. She became unable to raise her legs or flex her knees. Sensory loss was noted on the posterior aspect of both lower extremities and the anterior region below the knees. Myelography under fluoroscopy revealed dorsal contrast pooling (Figure 1A). Computed tomography (CT) myelography demonstrated a dorsal contrast defect from T7 to T12 in the sagittal view (Figure 1B) and axial compression of the thecal sac (Figure 1C).

**Figure 2:**
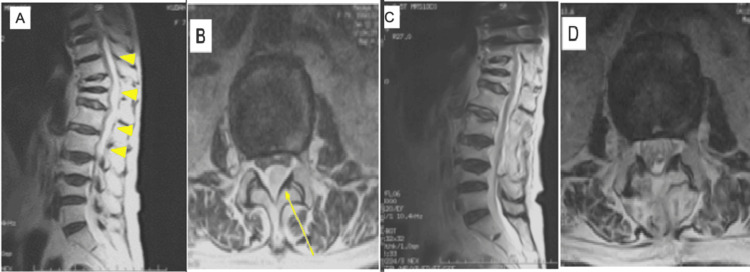
Pre- and postoperative MRI showing resolution of epidural hematoma in Case 1. (A) Preoperative sagittal T2-weighted MRI showing a hyperintense epidural mass extending from T6 to L4 (yellow arrowhead). (B) Preoperative axial T2-weighted MRI showing dorsal epidural hematoma compressing the thecal sac (yellow arrow). (C) Postoperative sagittal T2-weighted MRI demonstrating decompression of the spinal cord. (D) Postoperative axial T2-weighted MRI showing resolution of the hematoma and decompression of the spinal cord MRI: magnetic resonance imaging

Preoperative MRI showed a high T2- and low T1-intensity epidural mass extending from T6 to L4 on sagittal and axial views (Figures 2A, 2B), suggestive of an epidural hematoma. Postoperative MRI showed resolution of the hematoma and spinal cord decompression (Figures 2C, 2D).

Emergency decompression (T6-T8, L1-L3) and posterior fixation with titanium screws from T6 to T10 were performed six hours after symptom onset. No active bleeding point was identified intraoperatively. Blood loss was 880 mL, and transfusion was administered immediately postoperatively. Two hours after surgery, partial motor recovery was observed: MRC grades of 3/3 in the quadriceps, 2/1 in the tibialis anterior (TA), EHL, FHL, and gastrocnemius-soleus (GS) complex. By postoperative day 2, further improvement was noted: 5/4 in Q, 4/3 in TA, 3/3 in EHL, and 3/3 in FHL. After intensive rehabilitation, the patient regained independent ambulation using a T-cane, with full recovery of motor strength except 4/5 in the EHL. She was discharged three months after surgery.

Case 2

An 88-year-old woman presented with left lower extremity pain and numbness lasting four months. She had a history of L4/5 posterior lumbar fusion and decompression, though the current symptoms differed from her preoperative complaints. She was admitted to our hospital for further evaluation, including myelography. Her medical history included atrial fibrillation (on edoxaban), uterine fibroid, cataracts, hypertension, and diabetes mellitus. Laboratory tests showed a platelet count of 131,000/μL, PT-INR of 0.95, and APTT of 30.2 seconds. At the time of admission, she had no motor or sensory deficits. Straight leg raising tests were negative bilaterally, and no bladder or bowel dysfunction was noted. MRI showed left-sided lateral recess stenosis at L5/S1.

**Figure 3:**
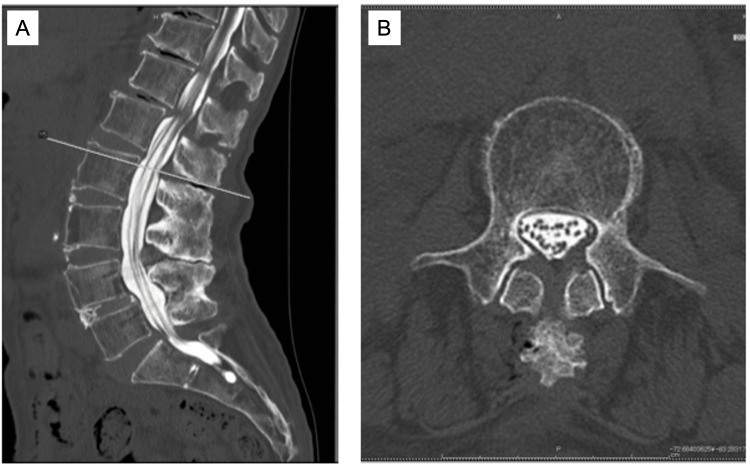
CT myelography images in Case 2. (A) Sagittal CT myelography performed immediately after contrast injection, showing no evidence of obstruction or hematoma. (B) Axial CT myelography showing preserved contrast flow without compression of the thecal sac CT: computed tomography

Myelography was performed on the day of admission using a 20-G needle. The patient was receiving edoxaban for high-risk atrial fibrillation. Given the elevated stroke risk, the decision was made to continue anticoagulation with close periprocedural observation. CT imaging obtained immediately after myelography showed no contrast defect in sagittal (Figure 3A) or axial views (Figure 3B). However, within an hour, the patient complained of pain and numbness in the left buttock and lateral thigh, followed by progressive motor weakness. Two hours after the procedure, her motor strength had deteriorated to MRC grades of 2/1 in the quadriceps, TA, GS, and 1/1 in the EHL and FHL. Sensory impairment was also noted, with diminished light touch and pinprick sensation on the posterior and anterior aspects of both lower legs.

**Figure 4:**
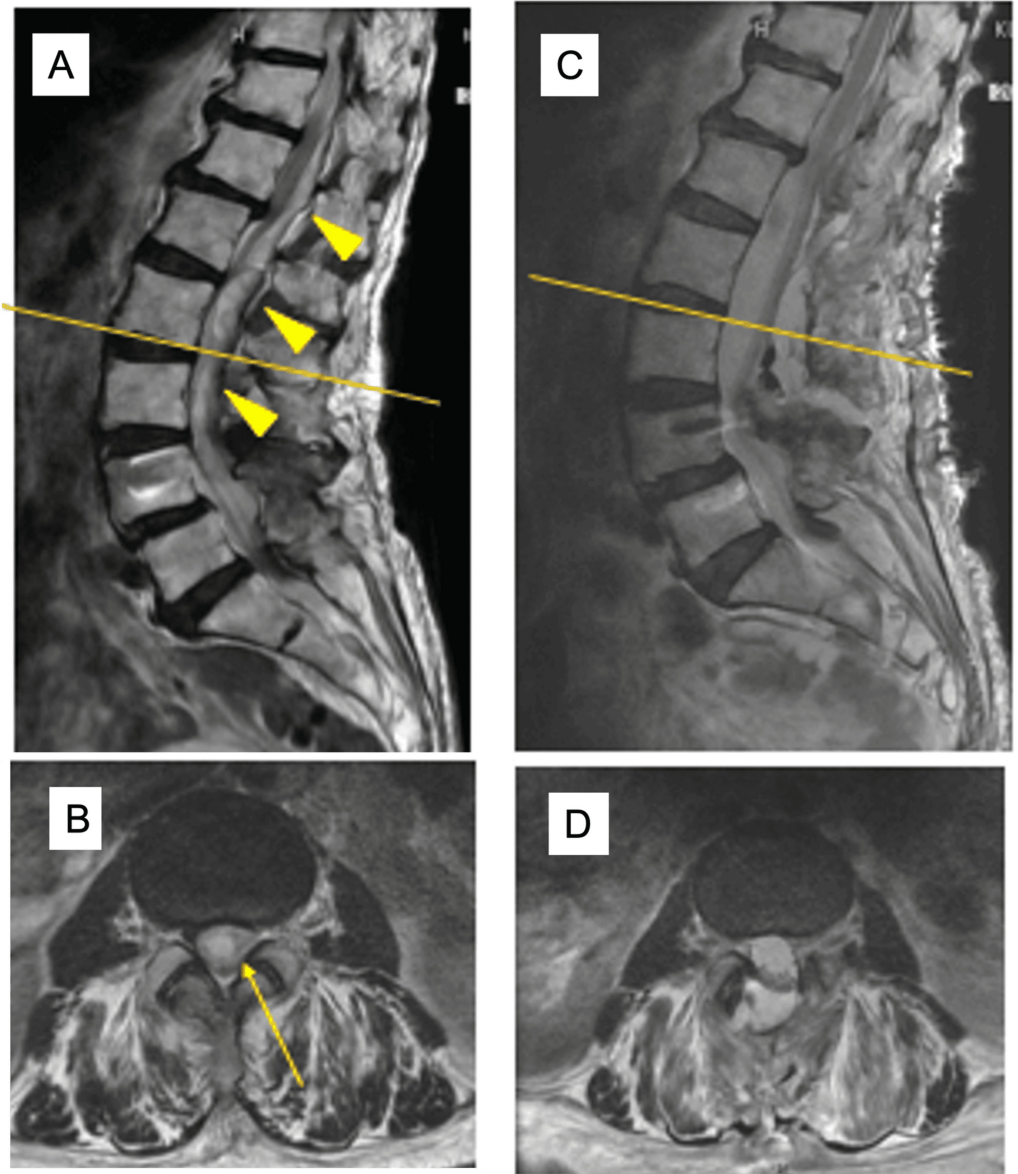
Pre- and postoperative MRI findings in Case 2. (A) Preoperative sagittal T2-weighted MRI demonstrating a hyperintense dorsal epidural mass extending from L1 to L4 (yellow arrowhead). (B) Preoperative axial T2-weighted MRI showing compression of the dural sac by epidural hematoma (yellow arrow). (C) Postoperative sagittal T2-weighted MRI confirming adequate decompression of the spinal canal. (D) Postoperative axial T2-weighted MRI revealing complete evacuation of the hematoma and decompression of neural elements MRI: magnetic resonance imaging

Preoperative sagittal and axial T2-weighted MRIs revealed a dorsal hyperintense epidural mass extending from L1 to L4 (Figures 4A, 4B). Postoperative MRIs showed complete evacuation of the hematoma and decompression of the spinal canal (Figures 4C, 4D).

Emergency decompression surgery was performed six hours after symptom onset. L1-L4 laminectomy was performed, and a large dorsal epidural hematoma was evacuated, although the bleeding source could not be identified. Motor function in the quadriceps improved immediately postoperatively (4/4), with the ability to elevate the knee. By postoperative day 2, motor strength improved to 5/5 in the iliopsoas and quadriceps, and 5/4 in the TA and GS. Following rehabilitation, she regained full strength (5/5) in all muscle groups and was able to walk independently without a cane. She was discharged one month after surgery.

## Discussion

CT myelography remains a valuable diagnostic modality in spine surgery, particularly for assessing dynamic or positional spinal stenosis [5]. Despite the widespread adoption of MRI, CT myelography continues to be routinely performed in our institution, where it contributes significantly to surgical decision-making. Complications are rare, reinforcing the safety profile of this modality. Among 13,259 CT myelography procedures conducted at our center between 2011 and 2023, only two cases of epidural hematoma occurred, an incidence of less than 0.01%.

Epidural hematoma following lumbar puncture or myelography is exceedingly rare but potentially devastating. Iatrogenic spinal hematomas have been documented in the literature, with a range of underlying etiologies including traumatic puncture, anticoagulant use, and coagulopathies [6,7]. Our observations are consistent with prior reports suggesting a possible association between antithrombotic therapy and the development of SEHs.Brown et al. reviewed 35 cases of iatrogenic spinal hematoma and emphasized that, although both surgical and conservative treatments yielded similar outcomes, early diagnosis and treatment were critical to recovery [6]. Several other case reports have described spinal hematomas following diagnostic lumbar puncture or spinal anesthesia [8-11]. In contrast, our report is the first to describe epidural hematoma following CT myelography performed without discontinuing antithrombotic agents, highlighting the importance of individualized periprocedural risk assessment.

Current guidelines suggest checking coagulation parameters such as PT and activated partial thromboplastin time (APTT) and withholding anticoagulant therapy before lumbar puncture to reduce the risk of hemorrhagic complications [4,12]. However, in clinical practice, approaches may vary depending on institutional protocols, urgency of testing, and patient comorbidities. In our institution, CT myelography is occasionally performed without discontinuing antithrombotic therapy, including aspirin and novel oral anticoagulants, after careful risk-benefit assessment and informed consent.

The timing of surgical intervention is a key prognostic factor. In a review by Rodrigues et al., delayed diagnosis beyond 12 hours was associated with poor neurological recovery and a higher likelihood of permanent deficits [13]. Conversely, when surgical decompression is performed promptly, neurological recovery is often favorable, even in patients with complete motor loss at onset. Our two cases support this, as both patients underwent emergency decompression within six hours of symptom onset and experienced substantial functional recovery.

Patient-specific factors, particularly the use of antiplatelet or anticoagulant agents, should be carefully considered. In our series, one patient was receiving aspirin and the other edoxaban. In both cases presented, antithrombotic therapy was continued due to compelling clinical indications. Case 1 involved aspirin therapy for secondary prevention following myocardial infarction and coronary stenting, while Case 2 involved edoxaban for high-risk atrial fibrillation. The prescribing physicians judged that the thromboembolic risks associated with discontinuation outweighed the potential bleeding risks. CT myelography was, therefore, performed under careful clinical and radiological monitoring.* *Antithrombotic agents have been frequently implicated in spontaneous and postprocedural spinal hematomas [2,6,10,14], highlighting the importance of individualized decision-making regarding periprocedural management.

This report has limitations inherent to case studies, including the small sample size and lack of generalizability. However, the rarity of the condition and the detailed clinical course presented herein offer valuable insights into management strategies for high-risk patients undergoing CT myelography. Although both patients recovered neurologically after timely surgical intervention, these cases highlight the potential risk of epidural hematoma in patients undergoing CT myelography while on antithrombotic therapy. The decision to continue such medications should be made with caution, considering both thromboembolic and bleeding risks, and only in settings where emergency intervention is readily available. General recommendations cannot be made based on these limited cases. Our findings underscore the importance of institutional preparedness and rapid access to neurosurgical care when undertaking spinal diagnostic procedures in high-risk populations.

## Conclusions

In our high-volume center, the incidence of epidural hematoma following CT myelography was exceedingly low (<0.05%). Both patients recovered fully after prompt decompression. These findings suggest that continuing antithrombotic therapy during CT myelography may be acceptable in select cases, provided that patients are carefully monitored and emergency intervention is readily available. Comprehensive preprocedure counseling remains essential.
